# Cardiac monitoring of dogs via smartphone mechanocardiography: a feasibility study

**DOI:** 10.1186/s12938-019-0667-9

**Published:** 2019-04-23

**Authors:** Olli Lahdenoja, Tero Hurnanen, Matti Kaisti, Juho Koskinen, Jarno Tuominen, Matti Vähä-Heikkilä, Laura Parikka, Maria Wiberg, Tero Koivisto, Mikko Pänkäälä

**Affiliations:** 1 Department of Future Technologies, Faculty of Science and Engineering, University of Turku, Vesilinnantie 5, 20014 Turku, Finland; 2 Department of Equine and Small Animal Medicine, Faculty of Veterinary Medicine, University of Helsinki, PL 57 Koetilantie 2, 00014 Helsinki, Finland

**Keywords:** Seismocardiography, SCG, Gyrocardiography, GCG, Mechanocardiography, MCG, Electrocardiography, ECG, Smartphone, Dog

## Abstract

**Background:**

In the context of monitoring dogs, usually, accelerometers have been used to measure the dog’s movement activity. Here, we study another application of the accelerometers (and gyroscopes)—seismocardiography (SCG) and gyrocardiography (GCG)—to monitor the dog’s heart. Together, 3-axis SCG and 3-axis GCG constitute of 6-axis mechanocardiography (MCG), which is inbuilt to most modern smartphones. Thus, the objective of this study is to assess the feasibility of using a smartphone-only solution to studying dog’s heart.

**Methods:**

A clinical trial (CT) was conducted at the University Small Animal Hospital, University of Helsinki, Finland. 14 dogs (3 breeds) including 18 measurements (about one half of all) where the dog’s status was such that it was still and not panting were further selected for the heart rate (HR) analysis (each signal with a duration of 1 min). The measurement device in the CT was a custom Holter monitor including synchronized 6-axis MCG and ECG. In addition, 16 dogs (9 breeds, one mixed-breed) were measured at home settings by the dog owners themselves using Sony Xperia Android smartphone sensor to further validate the applicability of the method.

**Results:**

The developed algorithm was able to select 10 good-quality signals from the 18 CT measurements, and for 7 of these, the automated algorithm was able to detect HR with deviation below or equal to 5 bpm (compared to ECG). Further visual analysis verified that, for approximately half of the dogs, the signal quality at home environment was sufficient for HR extraction at least in some signal locations, while the motion artifacts due to dog’s movements are the main challenges of the method.

**Conclusion:**

With improved data analysis techniques for managing noisy measurements, the proposed approach could be useful in home use. The advantage of the method is that it can operate as a stand-alone application without requiring any extra equipment (such as smart collar or ECG patch).

## Background

Pets (and dogs along) are part of the everyday life of millions of people worldwide. The purpose of this work is to study new ways to explore the health of dog’s heart, since it could be expected that dog owners especially would be tend to know the condition of their dogs [1–3]. Currently, the possibilities to access this information are very limited in out of clinic setting. Thus, measuring the well-being of the dog (e.g., for preventive purposes after exercise) could have a huge commercial market among the pet owners [4]. As with humans, also dogs may have different heart abnormalities and diseases which typical forms usually depend on the breed of the dog [5–8]. In the clinical settings at veterinary clinic, the cardiac measurements of the pet’s heart can be performed with standard ECG or with other modalities [4]. However, as for humans, sometimes, the pet owner may be unaware of the pet’s disease (or condition) and, therefore, does not acquire medical help from veterinary clinic even if it would be needed. Also the veterinary clinic might want to extend the monitoring of the pet to home settings for a longer time period after, e.g., a surgical operation.

As an extension to our previous work in using smartphones for detecting heart conditions in humans [9–11], in this work, we propose using accelerometers (and gyroscopes) to the monitoring of the dog’s heart rhythm. This is performed in both in clinical and home settings. A sensing device could be a smartphone or some other device attached to the dog’s collar or vest (also multiple sensors could potentially be used simultaneously) [12, 13]. 3-axis accelerometers (and 3-axis gyroscopes), i.e., 6-axis MCG, could be a feasible alternative as a sensing modality for pet monitoring, due to its non-invasive nature and potential tolerance to factors such as (slight) fur between the sensor and the dog’s skin (unlike ECG) and a low-power consumption in a longer follow-up sensing [12, 13]. To the best our knowledge, there has been very few prior works in studying the application of SCG or MCG to the animal health, except the recent work utilizing SCG for mice (anesthesia was used in that trial for the animals) [14].

The monitoring of dog’s heart at home settings can be performed, e.g., with portable ECG equipment [15–18]. For this purpose, one alternative is the AliveCor’s ECG, registered for animal studies. The AliveCor’s ECG, for instance, contacts the dog’s skin directly with two metal electrodes integrated into a small external package with usually no requirement of fur removal, which, in turn, communicates with a smartphone through an ultrasonic acoustic interface [19]. The usage of the accelerometers have been proposed for monitoring of dog’s activity (e.g., during some longer time period) [20, 21]. For instance, a smart dog collar can be used for making a summary of pet’s movements during a longer time period providing a summary of the different activities for the pet owner’s computer [12, 13].

Seismocardiography (SCG) is a well-known modality of measuring the heart’s motion using accelerometers attached on the chest [22–24]. It can be used to supplement ECG in situations where also the true mechanical motion of the heart is to be monitored, for instance in the form of deriving time-intervals or electromechanical delays. At the moment, the electromechanical delays are not extensively used in clinical practice, but this can change when better data are available. Ballistocardiography (BCG) measures the overall changes induced by the heart movements to the body, for example, using a smart weighting scale [25]. Gyrocardiography (GCG) is another, more recently proposed modality measuring the angular rate of the micromovements induced by heart to the chest using gyroscopes. It measures the rotational aspect of heart movement, which is in major role when the heart pumps blood to the circulatory system [9]. Recently, it was also shown that, using multiple sensors (SCG, GCG, and joint ECG) attached to the skin and a machine learning algorithm, it was possible to extract the cardiac parameters of a human even when running in a treadmill [26].

## Methods

### Animal study protocol and measurement set-up

For the clinical study, a permission was obtained from Research Ethics Committee of University of Helsinki’s Viikki Campus (statement no. 8/2016) and they were performed at the University Small Animal Hospital, University of Helsinki, Finland. In addition to the clinical trial, we performed additional data gathering from voluntary dog owners mainly among the staff of Department of Future Technologies, University of Turku, Finland. Each dog owner filled an informed consent regarding the participation to the study including the necessary instructions.

#### Clinical trial (*CT-A*)

**Fig. 1:**
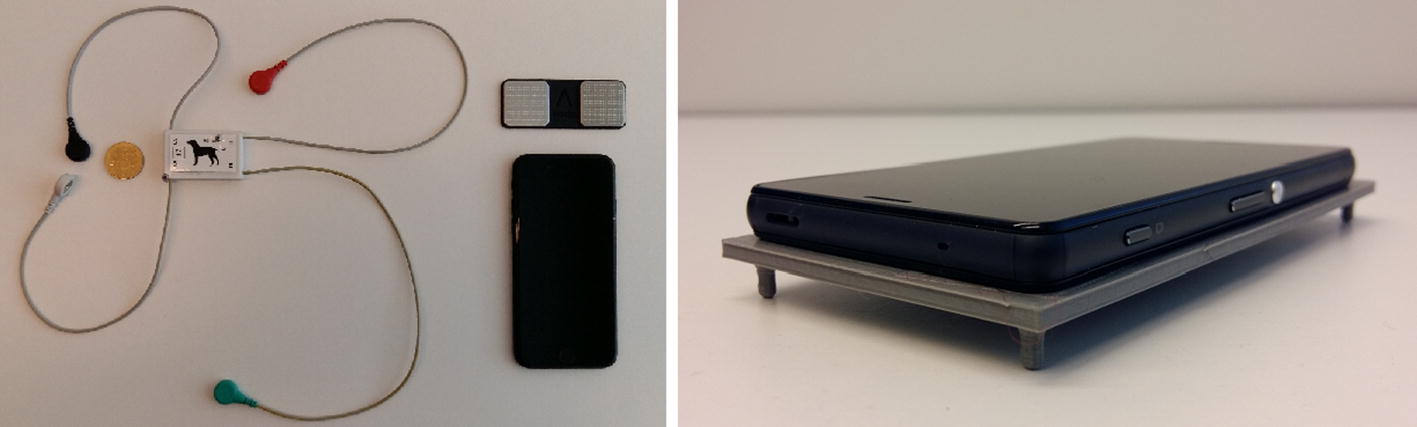
The available measurement devices of the study. The devices used in the measurements: (1) our custom Holter monitor (on the left, used in *CT-A*), (2) AliveCor’s phone ECG and patch (in the middle, used in *HM-C*), and (3) a 3D printed mounting for Android device (used in *HM-B*, not the cover, on the right) for improved contact and to avoid the sliding of the phone

Devices: For the clinical trial (*CT-A*), both ECG and IMU signal were gathered with the miniature sensor system reported in [9, 27]. The sensor system (“Holter”) (see Fig. 1) is built on an ARM Cortex M0-based microcontroller with Bluetooth capability to enable wireless control of the device. This feature can be also exploited in use cases, where IMU is located in a mobile phone, while the ECG acquisition is performed by the Holter device. In such cases, the mobile phone sets the real-time clock of the Holter device, enabling alignment of the data samples in the post-processing phase. However, in this work, we used the stand-alone version of the device. For stand-alone usage, as the Holter device integrates both IMU and ECG front end, an accurate time-domain synchronization between the accelerometer, gyroscope, and ECG samples without any additional effort can be achieved. The raw data samples are written to a memory card in proprietary frames, each containing a time stamp with 1 ms resolution. Together with a large memory capacity, the low-power design approach for the sensor enables measurement periods of even several days in length [27].

Methods: The Holter sensor (stand-alone version) was wrapped/hold on the left lower lateral side, over the heart region, while dog was standing or lying on the right side. Simultaneous 1-lead ECG and 6 degree-of-freedom IMU (3-axis SCG and 3-axis GCG, i.e., 6-axis MCG) signal of length of at least 1 min was captured. Dogs: In total, initially, 32 dogs from three breeds were measured (4 Whippet, 19 Doberman, and 9 Newfoundland dogs). After initial inspection of the signals, only the measurements which status was that the dog was not nervous or panting (the status recorded while acquiring the measurements) were selected for further processing. Purpose: To find out can SCG/GCG be used to measure dog’s heart with respect to an ECG reference.

#### Home measurements (*HM-B*)

**Fig. 2:**
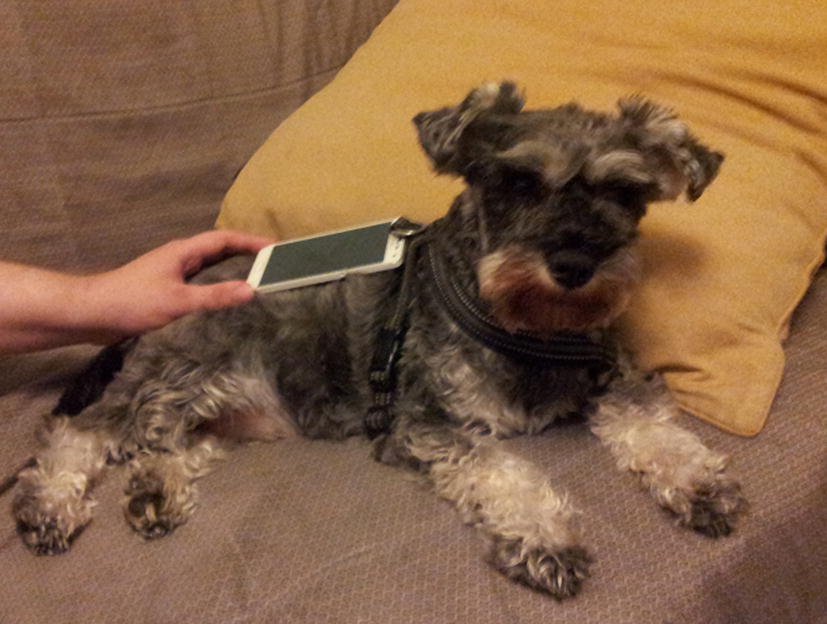
Implementing smartphone-only measurement. The placement of the smartphone in home measurements (*HM-B*) was usually on the ridge (while the dog was standing or in prone position) or on either lateral side of the dog, while the dog was resting on the other side. The users of the data collection application were advised to avoid grasping the phone hard. During the clinical measurements (*CT-A*), the Holter device was wrapped/hold on the left lower lateral side, over the heart region, while dog was standing or lying on the right side

Devices: The device was based on Sony Xperia series smartphone using Google Android OS as in [10, 11] (without ECG as reference). Methods: The dog owners themselves made the measurements. A data collection App was started and a button was pressed on the smartphone screen to start and end the measurement. The device was held on either lateral side or on the ridge of the, while the dog was in rest in a side, prone, or in a standing position (as shown in Fig. 2). Dogs: 16 dogs with total of 9 breeds (and 1 mixed-breed). Purpose: To test whether better signal quality could be obtained in home environment than at the veterinary clinic.

#### Home measurements (*HM-C*)

Devices: The second set-up for smartphone data collection was based on AliveCor’s Kardia while using the built-in IMU sensor of the iPhone running the AliveCor ECG simultaneously (i.e., capturing both ECG and MCG signal). Methods: The dog owners themselves made the measurements. The phone was held on the dog as in *HM-B* while simultaneously pressing the AliveCor patch usually to the paws of the dog. Dogs: Using this set-up, we obtained signals from only 3 dogs (Miniature Schnauzer, Maltese, and Wheaten Terrier), while, for two of these dogs, the simultaneous measurement did not succeed. Purpose: To test whether the smartphone IMU signal (and its important locations) collected from a dog can at least in some cases be directly mapped to smartphone ECG signal.

In practice, it was rather difficult in some cases to maintain short enough distance between the ECG patch and the smartphone to enable simultaneous ultrasonic communication and contact to dog’s skin while placing the smartphone simultaneously on the lateral side, on the ridge or on the abdomen of the dog and targeting that the dog was not moving. The sampling rate of the IMU sensor in the Android smartphone was 200 Hz and for the iPhone 100 Hz.

### Data analysis pipeline

The signal processing pipeline of *CT-A* starts by filtering each axis of the Holter signal (we used a fourth-order Butterworth band-pass filter). For smartphone-only case (trials *HM-B* and *HM-C*), the evaluations based on the signals (i.e., signal quality—how well the main SCG/GCG locations can be observed) were mostly made visually by a human expert. Different filters were tested and the best performing signal band in this case was 35–75 Hz. Although it is probable that most of the signal energy lies in lower frequency bands, the overall shape of the signal including more versatile set of dominant peak locations for peak detection was provided by the selected frequency band. The ECG signals and the IMU signals were resampled and aligned in time-domain to the same sampling frequency (of 200 Hz). Roughly 1 min length selection from each signal was finally chosen to the analysis (including 6-IMU axes and 1-lead ECG).

#### Automated axis and measurement selection

**Fig. 3:**
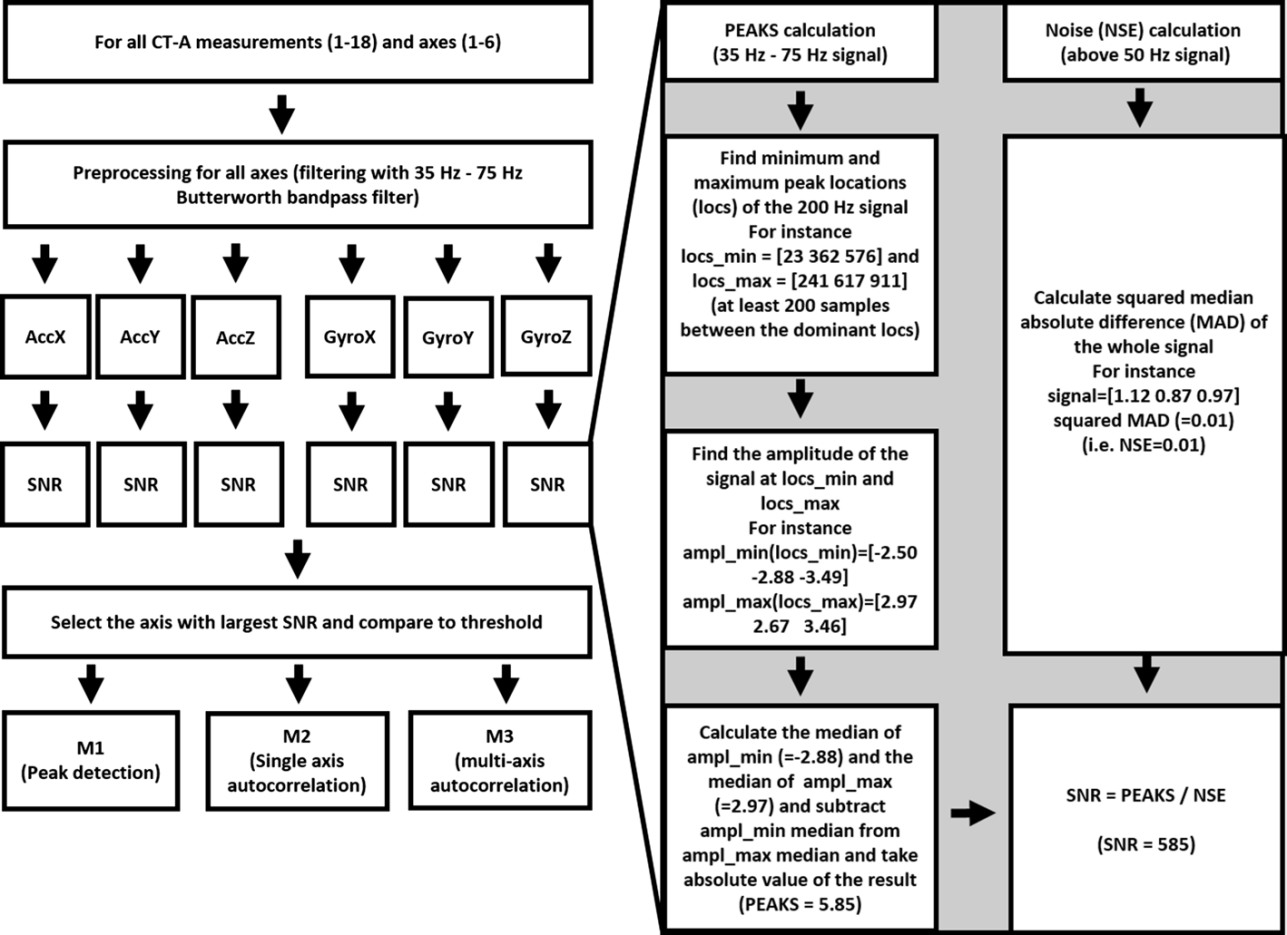
Axis and measurement selection method. A flowchart of the measurement and axis selection method considering trial *CT-A* and an example of SNR calculation (on the right)

The data analysis for *CT-A* starts with selecting the best IMU axis for further analysis (see Fig. 3). The selection of the best axis was accomplished axis-wise by the following procedure. First, a noise estimate (NSE) of the signal was obtained by high-pass filtering (above 50 Hz) the original signal with a 3th order Butterworth filter and calculating a squared mean absolute deviation (MAD) of the resulting signal’s amplitude. An estimate of the signal’s peak’s magnitudes was also calculated by first band-pass filtering (sixth-order Butterworth filter) the original signal in the frequency band of 35–75 Hz and extracting the peaks and the valleys from the signal with minimum peak distance of 1 s (the minimum spacing between successive detected peaks). The overall amplitudes of the resulting extreme points was then calculated through taking a median of the absolute values of the peak amplitudes, subtracted by the amplitudes of the valleys (PEAKS). Finally, as a signal quality estimating measure (SNR), the peak distance was divided by the noise estimate (SNR = PEAKS/NSE). The right side of Fig. 3 describes the SNR calculation [28]. The proposed method was considered sufficient for our application for the purpose of demonstrating that the axis selection and the selection of good-quality measurements could be beneficial.

We selected only the measurements which best axis’s SNR estimate was larger than a pre-determined threshold for the final heart rate (HR) estimation. As roughly half of the measurements were seen to be of sufficient quality, the threshold was in this case approximated accordingly. Of the total number of 18 signals selected to the evaluation, we this way (semi-automatically) identified 10 good-quality signals. However, by visual inspection by checking the correspondence of the dominant peaks, R-peaks in ECG, and AO peaks in SCG, even a better overall accuracy could have been obtained.

#### Heart rate estimation methods

We use three different methods for estimating the RR interval (or in fact AO–AO interval) to obtain the Dog’s HR from the IMU signals alone while using the miniature Holter device. It is first noted that the best axis on the basis of the SNR estimate is only selected for further processing in two of the three algorithms except one which uses all axes simultaneously. (M1) The first RR-interval estimation algorithm is very simple and it only locates the peaks with Matlab’s (R2017a) findpeaks function with minimum peak distance parameter set to 0.45 s (with maximum detectable HR slightly above 130 bpm). (M2) The second method is the same as reported in [29] for human atrial fibrillation (AFib) detection and it uses short autocorrelations to estimate HR for the selected best axis based on the SNR estimate (see the details in [29]). (M3) The third method is a multi-axis extension of the single-axis autocorrelation method presented in [30]. It can be estimated that the detected HR range in the case of autocorrelation algorithm is between 48 and 160 bpm which was seen sufficient for our application [29].

#### Measurement data selection

With regard to the clinical measurements (*CT-A*), 14 dogs and 18 measurements were selected to be used, while some of the measurements were repeated both in right lateral and standing positions. The measurements where the dog was not still or where the dog was panting were discarded in this analysis. As the dogs tended to be were quite nervous at the veterinary clinic, such a large portion (about one half) of the measurements were omitted. The dog’s breeds selected for HR estimation were as follows: Doberman (12), Newfoundland dog (1), and Whippet (1). The mean weight of the Dobermans was 32.8 kg with a standard deviation of 3.3 kg. The weight of the Newfoundland dog was 61 kg and the Whippets 17 kg. Eight of the measurements were performed in a right lateral position and ten in a standing position.

In the home settings (*HM-B*), we obtained measurements from 16 dogs with Android-based smartphone-only solution. The placement of the smartphone in the home measurements was usually on the ridge (while the dog was standing or in prone position) or on either lateral side of the dog, while the dog was resting on the other side (see Fig. 2). In this case, we applied a Butterworth band-pass filter with passband 1–45 Hz prior to the analysis to facilitate visual interpretation of the signals. The smallest dog among the home measurements was 7 kg Maltese and the largest dog was 35 kg Golden Retriever.

## Results

### The results of the clinical trial (*CT-A*)

The clinical trial results with different HR estimation algorithms (as described in the previous section) with ECG as a reference are reported in Table 1. A total of ten measurements from the 18 were selected to fulfill the quality criteria (the best axis’s SNR above a threshold, as described in the previous section). Each measurement used consisted of approximately 1 min measurement with joint ECG and IMU, while the total number of automatically detected beats (R-peaks) in ECG was 1685 (including all 18 measurements) of which only 18 (1.1%) were not detected correctly (confirmed by visual inspection). Thus, using an HR estimate obtained automatically from the ECG’s R peaks (as the median of all the individual time-differences between the beats) is accurate. In 7 of the 10 measurements of the best method (M2) in Table 1, the error (in bpm) is below or equal to 5, while, in one measurement (the last one), it is significantly larger. In Table 1 legend, also the breeds of the dogs in 10 measurements (corresponding to 9 dogs, one dog measured twice in two different positions) are described. The largest error was obtained for the Newfoundland dog. This is probably due to an inefficiency in the best axis (and best measurements) selection algorithm in selecting a good-quality measurement.

**Table 1:** The estimated HR results of the different methods (M1–3) and ECG ground truth HR regarding the clinical trial

(*CT-A*) selected measurement	#1	#2	#3	#4	#5	#6	#7	#8	#9	#10
Peak detection (HR) (bpm) (M1)	94	86	110	77	89	96	112	95	88	94
Deviation from ECG (in bpm)	28	0	47	3	20	2	5	8	4	44
Single-axis autocorrelation HR (bpm) (M2)	67	79	64	80	69	93	110	86	82	115
Deviation from ECG (in bpm)	1	7	1	0	0	5	7	1	2	23
6-axis autocorrelation HR (in bpm) (M3)	68	69	64	71	68	60	83	85	78	81
Deviation from ECG (in bpm)	2	17	1	9	1	38	34	2	6	57
ECG ground truth HR (bpm)	66	86	63	80	69	98	117	87	84	138

It can be observed that method M2 gives the lowest mean deviation from ECG. Measurements 1–2 and 4–9 are from Dobermans, while 1–2 are in a right lateral and 4–9 in a standing position. The measurements 1 and 5 are from the same dog. Measurements 3 and 10 are from Whippet and Newfoundland dog, respectively (both in a standing position)

### Smartphone-only home measurements (*HM-B*)

**Fig. 4:**
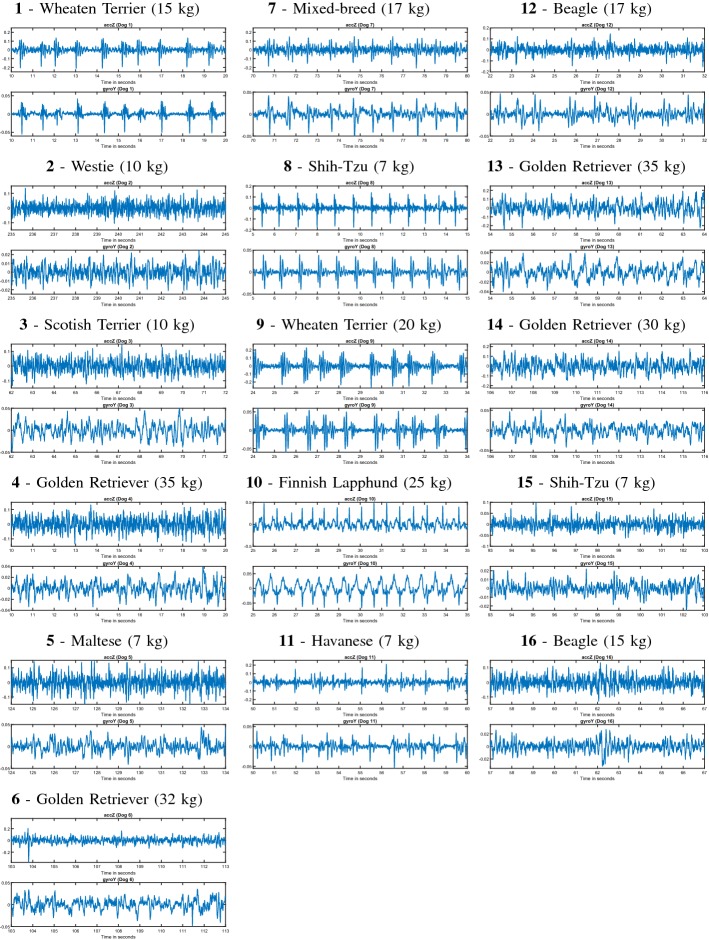
Examples of each signal measured at home. A good-quality signal segment (of a length of 10 s) according to human expert’s visual inspection from each of the 16 home measurements (of *HM-B*). Only the band-pass filtered AccZ and the GyroY signal axes are shown. The weights of the dogs are also shown in parenthesis. The signals were captured with the Android device while placing the smartphone on either lateral side of the dog or on the ridge, while in rest in a side, prone, or in a standing position

The quality of the home recordings was fairly good, as the dog owners were advised to obtain the measurements when the dog was relaxed and still. Still, some dog owners reported that the dog was initially not well accustomed with the measurement, and also some measurements were interrupted by dog changing position. In these cases, the length of the acquired signal may be shorter. However, it is probable that eventually (for most dogs) the smartphone measurement will more likely succeed after some attempts as the dog gets comfortable with the procedure. Figure 4 shows the IMU signals taken in these settings utilizing the Android device. The purpose was to extract visually best-quality 10 s duration segment from each dog. Each of the measurement plots in Fig. 4 correspond to the dog’s information in Table 2, where the breed, the weight, the age,and the overall number of measurements (which were in the end stored to the Android smartphone by the dog owners), can be seen. This number includes all the registered measurements by the dog owners, including potential unsuccessful measurements (e.g., because of aborting the recording due to dropping the phone). The estimated quality of each signal segment (with respect to how well the main signal peaks or HR can be identified) in Fig. 4 annotated by a human expert is also given in Table 2. For poor-quality signals, HR estimation would not probably work at all. In general, it can be observed that the heart beats are in about half of the cases clearly observable. While not all the dogs showed good signal quality, we estimate that, in about half or even most of the cases, HR could be extracted from the measurements (at least for some locations). Developing signal processing means to detect these locations automatically is, however, left for future work. This evaluation is based on visual characteristics of the signals, since, in this setting, we did not have an ECG reference available.

**Table 2:** The information of the dogs corresponding to the signals in Fig. 4 of the Android smartphone home measurements

*HM-B* #	Breed	Weight	Age	Tot. meas.	Quality
#1	Wheaten Terrier	15	12	4	Excellent
#2	Westie	10	8	5	Moderate
#3	Scotish Terrier	10	2	4	Poor
#4	Golden Retriever	35	9	1	Moderate
#5	Maltese	7	14	14	Moderate
#6	Golden Retriever	32	3	1	Moderate
#7	Mixed-breed	17	8	7	Excellent
#8	Shih-Tzu	7	4	4	Excellent
#9	Wheaten Terrier	20	9	3	Excellent
#10	Finnish Lapphund	25	3	4	Excellent
#11	Havanese	7	6	4	Good
#12	Beagle	17	4	1	Good
#13	Golden Retriever	35	5	1	Moderate
#14	Golden Retriever	30	2	1	Good
#15	Shih-Tzu	7	5	3	Moderate
#16	Beagle	15	2	2	Poor

Visually estimated signal quality of the signals in Fig.  4 made by human expert is also given. Due to motion artifacts, full signals were not evaluated in visual inspection

### Joint Alivecor’s ECG and smartphone-only measurement (*HM-C*)

**Fig. 5:**
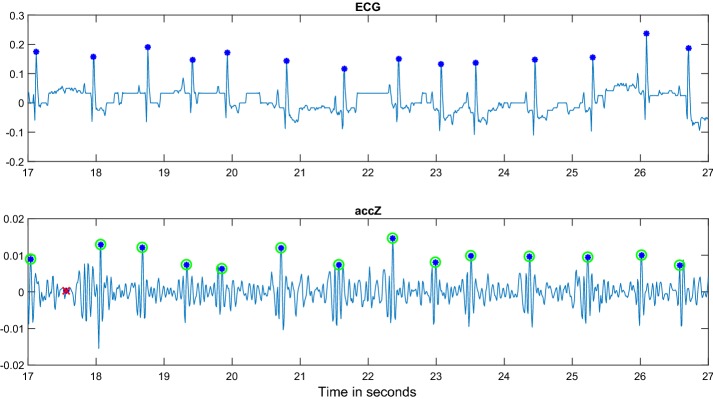
Example signal from 15 kg Wheaten Terrier. Time-aligned iPhone’s IMU signal (AccZ axis only shown below) and synchronized simultaneously captured ECG from AliveCor’s patch (above) converted from pdf output. It can be observed that the AO peaks in SCG match to the R peaks in ECG

As an another example with Alivecor’s ECG reference, a synchronized iPhone IMU signal and ECG taken in home setting is shown in Fig. 5. The ECG was digitized from the pdf output of the Alivecor’s ECG patch by a MATLAB script that we built. The smartphone’s ECG signal and iPhone IMU signal were further converted to the same sampling rate and subsequently aligned semi-automatically (i.e., shifted manually in time to the correct location). The signals (IMU and ECG) were acquired, so that the Alivecor’s patch was held in the paws of the dog and the phone simultaneously on the dog. While this experimental setting turned out too difficult for many dogs to obtain a good-quality recording (a few dogs tested), the acquired synchronized and time-aligned signal is a good example in that smartphone IMU can indeed be used to obtain both (a) the correct heart rhythm (verified against ECG) and (b) the individual R-peaks which correspond to the true AO peaks of the IMU. However, we were able to synchronize only one iPhone IMU + ECG signal in this setting, which was of sufficient quality for the final analysis. The collection of the IMU signal is much easier if simultaneous data acquisition with the Alivecor’s ECG patch is not required.

## Discussion

The purpose of this paper was to provide a feasibility evaluation of using an IMU-based smartphone-only solution to the measurement of dog’s health and well-being. As envisioned, the motion artifacts decrease the signal quality of MCG which is the most important limiting factor in this approach. However, when the dog is relaxed and while still it seems that the HR could be estimated for many different types of dogs (e.g., breeds and weights). A potential future application of this study could be a stand-alone smartphone application which could be used by dog owners to monitor the functioning of their pet’s heart for occasional check-up of dog’s heart condition. There could also be an option to store the results and track the trend in the dog’s resting HR in the long run.

It was noted during the clinical trials that the dog’s panting (which may occur especially if it is warm) affects to the acquired signal considerably. Therefore the user of a smartphone application should possibly be advised to perform the measurement only if the dog is still and not panting. Furthermore, the user should only gently hold the phone from sliding to make a successful acquisition of the dog’s heart signal. If this procedure is followed, also the probability of the sliding/dropping the phone is small. There are two issues that affect to the development of HR estimation algorithms for dogs. First, the range of the HR is wider for dogs (with varying breeds) than for humans [31]. Second, the breathing rate (BR) is higher for dogs (15–25 min^−1^ at rest). Therefore, it is, sometimes, difficult to differentiate the dog’s HR and BR from each other based on the MCG signal only.

As a limitation of this study, in the *CT-A* trial, there were only dogs from three breeds (mainly Dobermans). Thus, further work needs to be conducted to generalize our findings to all breeds. However, in the *HM-B* trial, nine breeds and one mixed-breed dogs were included. More than half of these had sufficient signal for HR estimation based on visual inspection. When comparing the different methods (M1–3, in Table 1), simple peak detection (M1) provides deviation of 5 bpm or less (from ECG) in half of the cases. For certain measurements with particularly low HR, it, however, fails. Single-axis autocorrelation (M2) performs best and it would seem to work both in low and high HR values. However, it does not work well for the dog number 10 (*CT-A*) which had the highest HR according to ECG. 6-axis autocorrelation (M3) had some difficulties among higher HR values. Its HR detection range is typically narrower, since it combines information from multiple axes HR estimates. If these differ from each other much, it may give a value in between the extreme values.

In general, our approach could be applied to monitor dog’s health even without anesthesia (which was used in [14] for mice). However, in the selection of the best measurements for HR estimation, the ratio of the measurements which succeeded was, in our case, known a priori. In the future, a more general algorithm should be developed that can blindly select only the good-quality measurements. However, as this work concerns feasibility evaluation only, this is left for future work. An another application which we did not consider in this work is heart rate variability (HRV) estimation. In addition to HR, HRV could be a potential indicator of dog’s wellness [4]. After successful HR extraction, it would be straightforward to extend our work towards HRV extraction.

The conducted clinical trial showed that it is possible to obtain the correct heart rhythm from dogs automatically using the proposed modality; however, the effect of artifacts caused, e.g., by panting or sinus arrhythmia needs to be taken into account. As the dogs in the veterinary clinic were quite nervous, a proper further validation procedure should be carried out using full 24 h Holter ECG recording, while simultaneously conducting occasional check-up measurements with the smartphone-only solution. Motion artifacts also limit the usage of the proposed method in the follow-up of dogs after a surgery and at least partly in using the method for the follow-up of the dogs’ recovery after exercise.

We also considered the effect of excessive dog’s fur to the non-invasive IMU sensing. At least in one of the home measurements (*HM-B*, dog number 10), it was reported by the dog owner that the dog had extensive fur. Despite this, the overall quality of the signal appears quite good. It seems obvious that excessive dog’s fur might, in some cases, still have a decreasing effect to signal quality, as our modality is based on either direct or indirect contacts to the dog’s fur/skin. During the work, we also developed a smart 3D printed casing (with four stick fingers) to avoid the effect of signal attenuation due to fur and the sliding of the phone. With regard to the main application, i.e., measuring the dog’s heart during still motion, it would be important that the applied smartphone solution (or application) would possess enough advanced methods for the signal-quality assessment, with an option to advice the user to redo the measurement if necessary. The implementational aspects of this are left to a future work.

In addition to our approach and ECG, there are also some other emerging modalities such as PPG (photoplethysmography) which could be utilized to monitor the dog’s vital signs [32]. While MCG recording measures the heart-induced mechanical activity, PPG measures optically the blood volume in the microvascular bed of tissue [32]. Intuitively, PPG could partly suffer from similar drawbacks than our method in that finding a proper sensing location from the dog could be an issue if there are excessive fur. About a question, whether our approach could potentially replace Alivecor’s ECG patch in clinical practice, the answer is probably no. On the other hand, if the dog owner’s intention was to only occasionally investigate the resting HR, potentially as a fitness/health indicator (and/or possibly BR in home settings), our approach in the form of a smartphone application could be a viable solution, since it does not require buying any extra equipment.

## Conclusion

It is concluded that there are several limitations which make the usage of this new modality challenging, but, with improved data analysis techniques mainly for managing noisy measurements, the proposed smartphone-only solution for measuring dog’s HR could still be useful in home use.
